# Prevalence and clinical features of celiac disease in patients with autoimmune thyroiditis: cross-sectional study

**DOI:** 10.1590/1516-3180-2014-1326725

**Published:** 2014-09-02

**Authors:** Aline Ventura, Marcelo Fernando Ronsoni, Maria Beatriz Cacese Shiozawa, Esther Buzaglo Dantas-Corrêa, Maria Heloisa Busi da Silva Canalli, Leonardo de Lucca Schiavon, Janaína Luz Narciso-Schiavon

**Affiliations:** I Medical Student. Núcleo de Estudos em Gastroenterologia e Hepatologia (NEGH), Universidade Federal de Santa Catarina (UFSC), Florianópolis, Santa Catarina, Brazil; II MD, MSc. Volunteer Staff, Department of Internal Medicine, Universidade Federal de Santa Catarina (UFSC), Florianópolis, Santa Catarina, Brazil; III MD, MSc. Professor, Department of Pathology, Universidade Federal de Santa Catarina (UFSC), Florianópolis, Santa Catarina, Brazil; IV MD, PhD. Adjunct Professor, Department of Internal Medicine, Gastroenterology Division, Universidade Federal de Santa Catarina (UFSC), Florianópolis, Santa Catarina, Brazil; V MD, MSc. Attending Physician, Endocrinology Service, "Polydoro Ernani de São Thiago" University Hospital, Universidade Federal de Santa Catarina (HU-UFSC), Florianópolis, Santa Catarina, Brazil

**Keywords:** Celiac disease, Thyroiditis, autoimmune, Transglutaminases, Biopsy, Hashimoto disease, Doença celíaca, Tireoidite autoimune, Transglutaminases, Biópsia, Doença de Hashimoto

## Abstract

**CONTEXT AND OBJECTIVE:**

Celiac disease is an autoimmune disorder with an average prevalence of 1% in Europe and the United States. Because of strong European ancestry in southern Brazil, this study aimed to evaluate the seroprevalence of celiac disease among autoimmune thyroiditis patients.

**DESIGN AND SETTING:**

Cross-sectional study in a public university hospital.

**METHODS:**

This cross-sectional prevalence study included autoimmune thyroiditis patients who were tested for anti-endomysial and anti-transglutaminase antibodies between August 2010 and July 2011.

**RESULTS:**

Fifty-three patients with autoimmune thyroiditis were included; 92.5% were women, with mean age of 49.0 ± 13.5 years. Five patients (9.3%) were serologically positive for celiac disease: three of them (5.6%) were reactive for anti-endomysial antibodies and two (3.7%) for anti-transglutaminase. None of them exhibited anemia and one presented diarrhea. Endoscopy was performed on two patients: one with normal histology and the other with lymphocytic infiltrate and villous atrophy.

**CONCLUSION:**

The prevalence of celiac disease among patients with autoimmune thyroid disease was 9.3%; one patient complained of diarrhea and none presented anemia. Among at-risk populations, like autoimmune thyroiditis patients, the presence of diarrhea or anemia should not be used as a criterion for indicating celiac disease investigation. This must be done for all autoimmune thyroiditis patients because of its high prevalence.

## Abstract (pt)

**CONTEXTO E OBJETIVO:**

A doença celíaca é uma doença autoimune, com prevalência média de 1% na Europa e nos Estados Unidos. Em função da forte ascendência europeia no sul do Brasil, este estudo objetiva relatar a soroprevalência de doença celíaca em indivíduos com tireoidite autoimune.

**TIPO DE ESTUDO E LOCAL:**

Estudo transversal em um hospital público universitário.

**MÉTODOS:**

Este estudo transversal de prevalência incluiu pacientes com tireoidite autoimune que foram submetidos a testes de anticorpos antiendomísio e antitransglutaminase entre agosto de 2010 e julho de 2011.

**RESULTADOS:**

Foram incluídos 53 pacientes com tireoidite autoimune, 92,5% mulheres, com idade média de 49,0 ± 13,5 anos. Cinco (9,3%) pacientes apresentaram sorologia positiva para doença celíaca, sendo três (5,6%) com anticorpo antiendomísio positivo e dois (3,7%) com antitransglutaminase positivo. Nenhum paciente apresentou anemia e um apresentou diarreia. Apenas dois pacientes realizaram endoscopia: um com histologia normal e outro apresentou infiltrado linfocitário e atrofia vilositária.

**CONCLUSÕES:**

A prevalência de doença celíaca entre pacientes com doença autoimune da tireoide foi de 9,3%; um paciente queixou-se de diarreia e ninguém apresentou anemia. Em populações de risco, como é o caso de pacientes com tireoidite autoimune, a presença de diarreia ou anemia não devem ser utilizados como critério para indicar investigação de doença celíaca, que deve ser feita em todos os indivíduos com tireoidite autoimune devido a sua alta prevalência.

## INTRODUCTION

Celiac disease is an autoimmune disorder characterized by permanent intolerance to ingested gluten.^1^ Once considered rare, celiac disease has a prevalence of approximately 1% in Europe and the United States.^2^ In Brazil, the seroprevalence amongst blood donors ranges from 1:417 in the south, to 1:214 - 1:286 in São Paulo, and 1:681 in Brasília, possibly explained by the European origin of the population of southern Brazil.^3^^-^ 6

Celiac disease is characterized by chronic inflammation of the mucosa of the proximal small intestine, which improves when foods containing gluten are excluded from the diet and relapses when these foods are reintroduced. Structurally, the main features consist of villous atrophy in the small intestine, crypt hyperplasia and inflammatory infiltration of the lamina propria and intraepithelial compartments. The functional changes include reduction in food digestion, decreased absorption of macro and micronutrients and increased secretion of water and solutes.^7^ Celiac disease may remain asymptomatic throughout life, or manifest clinically at any time, with various clinical presentations, such as iron deficiency anemia, chronic diarrhea, abdominal pain or discomfort, weight loss, neurological symptoms, dermatitis herpetiformis, hypoproteinemia, hypocalcaemia and elevated liver enzymes, among others.^8^^-^ 10 However, it is increasingly recognized that symptomatic cases represent only the tip of the celiac iceberg.^10^^-^ 12 Asymptomatic patients often remain undiagnosed, since screening tests are usually carried out only on patients with typical manifestations of the disease.^11^ Undiagnosed celiac disease has been correlated with a fourfold increase in the risk of death.^12^

The screening tests include serological tests for anti-endomysial (EMA) and tissue anti-transglutaminase (TTG) immunoglobulin A (IgA) antibodies, which, when positive, are indicative that the patient presents the disease. Although these tests are not conclusive, since histological confirmation is required, investigation of EMA and TTG antibodies is a useful and less invasive tool that enables screening of a great number of people. Thus, these tests can ensure that either asymptomatic individuals or those with mild clinical forms are identified.^13^^,^ 14 Patients who are serologically positive for celiac disease and normal histology present its latent form.^7^

Recently, strong associations between celiac disease and various autoimmune diseases, including autoimmune thyroid disease, type 1 diabetes mellitus, primary biliary cirrhosis, inflammatory bowel diseases and autoimmune adrenal insufficiency, have been demonstrated. It is believed that 2-5% of patients with autoimmune thyroid disorders have celiac disease, due to common genetic characteristics (HLA-DQ2 and HLA-DQ8).^15^^,^ 16

## OBJECTIVE

Because of the strong European origin in the state of Santa Catarina, it would be natural to assume that the prevalence of celiac disease in the south of Brazil is significantly higher than that found in other regions of the country. Considering the association between celiac disease and autoimmune diseases, and the silent spectrum of the disease, this study aimed to investigate the prevalence of celiac disease in individuals with autoimmune thyroiditis and to describe their clinical features.

## METHODS

### Patients

This descriptive cross-sectional prevalence study included all consecutive adult patients with autoimmune thyroid disease seen at the Endocrinology Outpatient Clinic of the University Hospital of the Federal University of Santa Catarina between August 2010 and July 2011. They underwent celiac disease screening for this study through investigation of EMA and TTG antibodies, after giving their written informed consent. Patients without complete clinical and laboratory data registration in their medical records were excluded from the study. The study protocol conformed to the ethical guidelines of the 1975 Helsinki Declaration and was approved by our institutional review board under the number 857/10.

### Methods

Assuming an estimated prevalence rate of 11% for celiac disease among patients with autoimmune thyroiditis^17^ and using a 95% confidence interval (CI) and an accuracy of 0.8%, the estimated sample size was 50 individuals.^18^

At outpatient consultations, an interview guided by a specific protocol designed for this study was conducted in order to outline clinical features and investigate the presence of gastrointestinal symptoms among patients with a previous diagnosis of autoimmune thyroiditis. The diagnostic criteria for autoimmune thyroiditis comprised positivity for antithyroglobulin (ATG) and antithyroid peroxidase (ATPO) antibodies and altered serum thyroid stimulating hormone (TSH) or a positive ultrasound examination.^19^^,^ 20 Clinical, laboratory and histological findings were collected from the data contained in the medical records. Patients were analyzed according to the following characteristics: age, gender, race, family history, diarrhea, body mass index, antinuclear antibody (ANA), free thyroxin (FT4), thyroid-stimulating hormone (TSH), hemoglobin, serum iron, serum ferritin and transferrin saturation. 

The IgA TTG and IgA EMA serological tests were applied for celiac disease screening.^2^^-^ 4 ^,^ 7 EMA was detected by means of indirect immunofluorescence on monkey esophagus tissue (Viro-Immun Labor Diagnostika, Germany) and TTG was detected using a commercial enzyme-linked immunosorbent assay (ELISA) with rabbit polyclonal antiserum (OrgentecDiagnostika GmbH, Germany). Patients with either EMA or TTG-reactive antibodies were considered to be serologically positive for celiac disease.^21^ Biochemical tests were expressed as absolute values. Those who presented negative results for both antibodies underwent quantitative assaying of total serum IgA, which ruled out a selective IgA deficiency.

Upper digestive endoscopy and duodenal biopsy was indicated for individuals who were serologically positive for celiac disease. Duodenal fragments were fixed in 10% formalin, processed with paraffin and stained with hematoxylin-eosin (HE). For diagnosing celiac disease, the following histological variables were analyzed: lymphocytic infiltrate, villous atrophy and crypt hyperplasia.

### Statistical analysis

Continuous variables were compared using the Student t test or the Mann-Whitney test when appropriate. Categorical variables were compared using the chi-square or Fisher exact test. P values less than 0.05 were considered statistically significant. All tests were two-tailed and were conducted using the Statistical Package for the Social Sciences (SPSS, Chicago, IL, USA), version 17.0.

## RESULTS

### Patients' characteristics

**Figure 1:**
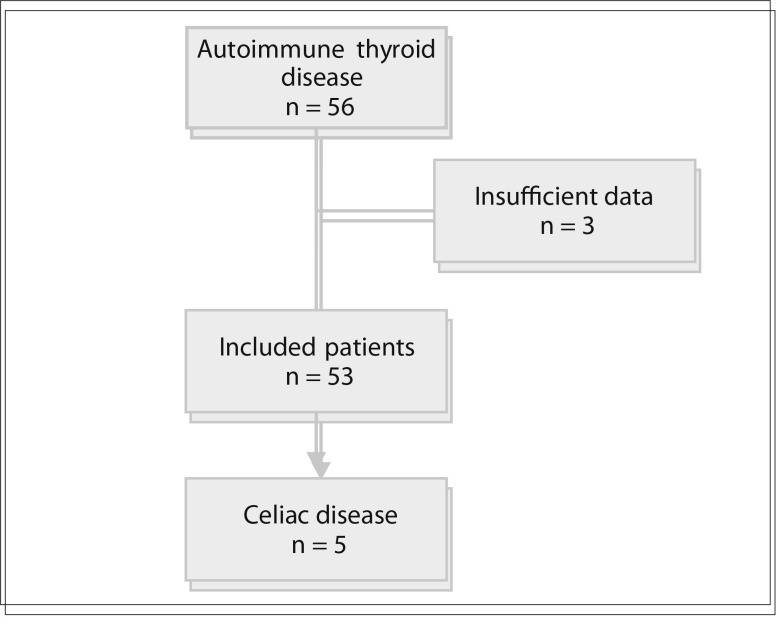
Flow diagram of the potential candidates for participation in the study, reason for exclusions and subjects enrolled.

Between August 2010 and July 2011, 56 patients with autoimmune thyroid disease were considered for enrollment. Three individuals were excluded from the study because they were not tested for EMA and TTG antibodies (Figure 1).

The characteristics of the 53 consecutive patients fulfilling the entry criteria are summarized in Table 1. Their mean age was 49.0 ± 13.5 (median 51.0) years; 92.5% were women and 87.8% were Caucasian. None of the patients included presented IgA deficiency.

**Table 1:** Clinical and laboratory characteristics of 53 patients with autoimmune thyroiditis according to the presence of celiac disease (CD) antibodies: anti-endomysial and/or anti-transglutaminase

Characteristics	Total	CD (+) n = 5	CD (-) n = 48	P^*^
Age (years)^†^	51.0	21.0	53.0	0.030
Female gender (%)	92.5	60.0	95.8	0.040
Caucasian (%)	87.8	80.0	88.6	0.495
Positive family history (%)	0.0	0.0	0.0	-
Diarrhea (%)	13.2	20.0	12.5	0.522
BMI (kg/m^2^)^†^	26.8	26.1	27.1	0.285
ANA positive^†^	12.0	20.0	11.1	0.487
Free thyroxin (ng/dl)^†^	1.2	1.2	1.2	0.617
TSH (mU/l)^†^	3.1	2.6	3.4	0.351
Hemoglobin (g/dl)	13.4	14.8	13.4	0.007
Serum iron (µg/ml)	73.8 ± 27.8	59.2 ± 22.6	74.4 ± 28.6	0.260
Serum ferritin (ng/ml)	65.5	28.3	65.5	0.475
Transferrin saturation (%)	20.0	16.0	20.0	0.212

* Student t test, Fisher's exact test or Mann-Whitney test, when appropriate, for comparison of the groups

† Median

BMI = body mass index

ANA = antinuclear antibody

TSH = thyroid stimulating hormone.

### Evaluation of patients who were serologically positive for celiac disease

**Figure 2:**
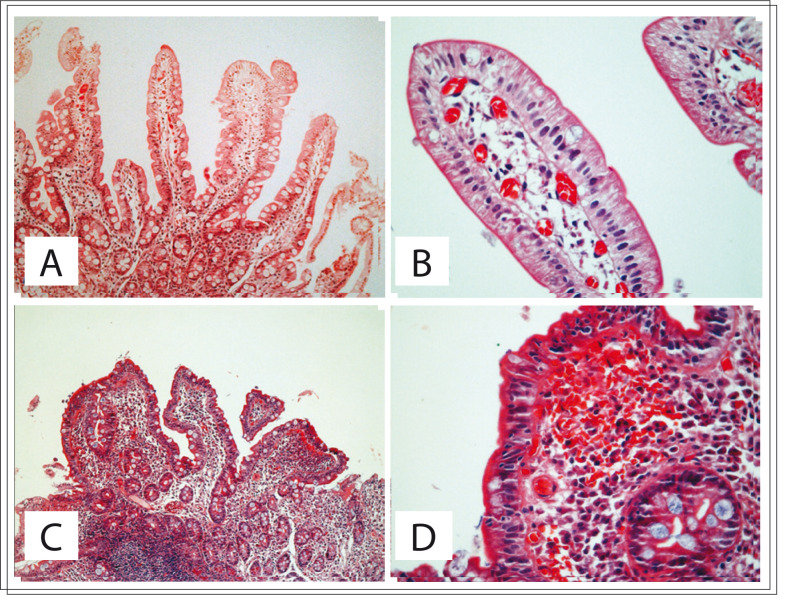
Distal duodenal biopsy exhibiting mucosa with normal histological pattern (A, B) and biopsy showing histological changes compatible with celiac disease (C, D). (A) Fragment of duodenal mucosa showing conserved architecture with long parallel fingerlike villi; usual leukocyte population of the lamina propria (hematoxylin-eosin, HE; 40 x). (B) Detail of the villus epithelium permeated by few lymphocytes, lamina propria with congested capillaries (HE; 400 x). (C) Duodenal mucosa showing significant inflammatory changes and intense structural villous shortening with edema, congestion and increased inflammatory cells in the lamina propria (HE; 40 x). (D) Detail of the duodenal villus with increased population of intraepithelial lymphocytes, plasma cells and many red blood cells in the lamina propria (HE; 400 x).

Five patients out of 53 (9.3%) were serologically positive for celiac disease (Table 2): three of them (5.6%) with reactive EMA antibodies and two (3.7%) with reactive TTG. None of the subjects demonstrated reactivity for both antibodies. None of them presented anemia, but most of the individuals showed transferrin saturation below the normal range (< 20%) and all of them showed low serum ferritin levels. Only one patient complained of diarrhea. Three patients were overweight. Only two patients who were serologically positive for celiac disease underwent upper digestive endoscopy. With regard to the histological findings from duodenal biopsy, one patient had normal histology (Figure 2A and 2B) and the other exhibited lymphocytic infiltrate, villous atrophy and crypt hyperplasia (Figure 2C and 2D).

**Table 2:** Clinical and laboratory characteristics of five individuals with autoimmune thyroiditis who were serologically positive for celiac disease

	Case 1	Case 2	Case 3	Case 4	Case 5
Age (years)	22	74	20	19	21
Gender	Female	Male	Female	Male	Female
Caucasian	Yes	No	Yes	Yes	Yes
BMI (kg/m^2^)	19.5	25.2	26.1	26.5	26.8
Diarrhea	No	Yes	No	No	No
Hemoglobin (g/dl)^*^	14.6	14.8	13.4	14.9	14.8
Serum ferritin (ng/ml)^†^	26	196	28	103	16
Transferrin saturation (%)‡	27	9	15	16	19
Anti-endomysial antibodies	(+)	(+)	(+)	(-)	(-)
Anti-transglutaminase	(-)	(-)	(-)	(+)	(+)
Underwent UDE	Yes	No	No	Yes	No
- lymphocytic infiltrate	No	-	-	Yes	-
- villous atrophy	No	-	-	Yes	-
- crypt hyperplasia	No	-	-	Yes	-

BMI = body mass index

UDE = upper digestive endoscopy

(+) = reactive

(-) = not reactive

* Reference value (RV) = 13 g/dl (♂); 12 g/dl (♀)

† RV = 250 μg/l (♂); 120 μg/l (♀)

‡ RV = 20-50%.

### Factors associated with serologically positive findings of celiac disease

When five individuals who were serologically positive for celiac disease were compared with 48 individuals with autoimune thyroid disease and negative antibodies for celiac disease, the positive individuals presented a lower median age (21.0 versus 53.0 years; P = 0.030), as well as a lower proportion of females (60.0% versus 95.8%; P = 0.040) and higher median hemoglobin (14.8 versus 13.4 g/dl; P = 0.007). No difference was observed with regard to race, family history of celiac disease, symptoms of diarrhea, body mass index (BMI) or positive ANA, FT4, TSH, serum iron, serum ferritin or transferrin saturation (Table 1).

## DISCUSSION

Mass screening for celiac disease is not supported by the present evidence.^22^ Currently, the best approach is to focus on case findings by screening individuals who are known to have a higher risk of celiac disease.^20^

The commercially available diagnostic tests for celiac disease are antigliadin antibody (AGA), EMA and TTG. In comparison with the other tests, AGA presents lower specificity (< 90%), and hence its use is no longer recommended in clinical practice. EMA and TTG IgA antibodies present high sensitivity (97-90%) and specificity (99-95%) for the serological diagnosis of celiac disease.^5^ It is well recognized that the combined use of the two antibodies (EMA and TTG) provides greater sensitivity in diagnosing celiac disease, since almost one third of the patients present reactivity for only one of the two antibodies.^23^ However, from a practical standpoint, the sensitivity of commercial assays may vary and does not necessarily represent what is described in the literature, especially in subjects with major villous atrophy.^24^

IgA deficiency is recognized as a cause of false negative serological results, and this is more common among individuals with celiac disease than in the general population (1:40 versus 1:400). Therefore, IgA should be assayed in individuals with no reactivity for either EMA or TTG IgA antibodies. If IgA deficiency is confirmed, individuals should undergo the TTG IgG test.^5^^,^ 25 None of the patients in this study who were serologically negative for celiac disease presented IgA deficiency; therefore, TTG IgG tests were not performed.

Thyroid hormones have an effect on the gastrointestinal tract at all levels of organization and clinicians have long recognized the associations that exist between gastrointestinal symptoms and thyroid disease. In cases of established hypothyroidism, the intestine may show marked structural alterations, and diffuse myxedematous infiltration is occasionally seen throughout the bowel wall. Sluggish motility in hypothyroidism cases may lead directly to constipation, atony and even intestinal obstruction.^26^

An association between celiac disease and autoimmune thyroiditis has been reported worldwide, especially in Europe. Increased prevalence of thyroid dysfunction has been reported in patients with celiac disease and vice versa. These thyroid disorders are sometimes diagnosed before, and sometimes after diagnosing celiac disease and beginning the gluten-free diet. Volta et al. reported a hypothyroidism rate of 5.7% among 70 celiac patients,^27^ similarly to Midhagen et al., who reported a prevalence of 5.8%.^28^ Velluzzi et al. evaluated 47 patients with celiac disease and 91 healthy controls, and found that the prevalence of ATPO was higher in celiac patients (29.7%) than in healthy controls (9.6%) (P < 0.001) and that thyroid echography frequently displayed a hypoechogenic pattern (42.5%). Five ATPO-positive celiac patients presented hypothyroidism (two overtly and three subclinically).^29^ Elfström et al. identified 15,439 individuals with celiac disease who had been diagnosed between 1964 and 2003. Of these, 1.0% presented hyperthyroidism, 2.4% had hypothyroidism, and 0.3% had thyroiditis before or after receiving the diagnosis of celiac disease.^30^ In Brazil, among 52 patients with celiac disease, 19.2% presented clinical hypothyroidism.^31^ Meloni et al. assessed 324 children with biopsy-proven celiac disease and observed that 10.5% developed autoimmune thyroid disease: 11 at the onset of celiac disease and 23 while under a gluten-free diet, with higher prevalence than among controls.^32^ Recently, Metso et al. evaluated 27 individuals with newly diagnosed celiac disease and investigated the presence of autoimmune thyroid diseases at the time of diagnosis and after one year on a gluten-free diet. At the time of diagnosis, there were more manifest (n = 7) or subclinical (n = 3) cases of thyroid disease among the celiac disease patients than among the controls (10/27 versus 3/27; P = 0.055). During the follow-up, the thyroid volume decreased significantly in the patients with celiac disease, compared with the controls, thus indicating progression of thyroid gland atrophy despite the gluten-free diet.^33^ Among 152 patients with celiac disease, Collins et al. reported that 14.5% had concomitant hypothyroidism. The mean levothyroxine dose before treatment for celiac disease and the weight-based levothyroxine dose needed to maintain a euthyroid state were higher in the cases than in 200 controls (154 µg versus 106 µg; P = 0.007). The doses decreased significantly after treatment of celiac disease (154 µg versus 111 µg; P = 0.030).^34^ Since it has been demonstrated that celiac disease may be present in patients with hypothyroidism requiring elevated levothyroxine doses, routine screening for celiac disease has been widely proposed for patients with autoimmune thyroid disease.^17^^,^ 34 ^,^ 35

In a study evaluating the presence of celiac disease among individuals with autoimmune thyroiditis, Sari et al. reported that among 101 Turkish children with autoimmune thyroiditis, 7.9% were positive for IgA TTG. Among five of these individuals who underwent duodenal biopsy, three presented marked subtotal villous atrophy whereas the other two showed normal histology.^20^ Recently, Merhdad et al. showed that there was low prevalence of positive serological tests for celiac disease (AGA IgA, EMA IgA, and TTG IgA) among 454 individuals with hypothyroidism in Iran. Only eleven patients (2.4%) were serologically positive for celiac disease, and two patients with documented villous atrophy were diagnosed with classic celiac disease (0.4%).^21^ In the Netherlands, Hadithi et al. found that sixteen (15%) out of 104 patients with Hashimoto's thyroiditis were positive for one or a combination of four celiac serological tests (AGA IgA/IgG, EMA and TTG). Five patients (4.8%) presented either intraepithelial lymphocytosis or crypt hyperplasia and villous atrophy in small intestine biopsies, in association with gastrointestinal symptoms (diarrhea or abdominal pain) and were therefore diagnosed with classic celiac disease.^36^

In Finland, Collin et al. assessed 83 patients with autoimmune thyroiditis who were tested for EMA IgA and AGA IgA/IgG, and found three asymptomatic celiac patients, and one patient with previously diagnosed celiac disease, i.e. an overall frequency of 4.8%.^37^ A study in northern Brazil showed that among 457 individuals with antithyroid antibodies, 2.2% were reactive for EMA IgA and 3.1% for TTG.^38^

Many studies have been conducted in Italy on this subject, and the prevalence of celiac disease reported among individuals with autoimmune thyroid disease varies enormously. Iuorio et al. described a high prevalence of EMA IgA/immunoglobulin G (IgG) antibodies (27.4%) among 113 untreated patients with autoimmune thyroiditis,^39^ while Ravaglia et al. reported that the prevalence of celiac disease antibodies (EMA IgA, TTG IgA and AGA IgG in the presence of IgA deficiency) was 1.5% among 737 individual with autoimmune thyroiditis.^15^ Intermediate prevalence has been described by other authors such as Cuoco et al., who evaluated 92 patients with autoimmune thyroid disease, of whom 4.3% were positive for AGA and EMA and had celiac disease.^40^ Valentino et al. screened 150 patients with autoimmune thyroid disease for celiac antibodies (EMA IgA). Five patients (3.3%) were EMA-reactive and all of them exhibited subtotal villous atrophy in jejunal biopsies, thus allowing the diagnosis of celiac disease.^41^ Berti et al. reported that the prevalence of celiac disease (EMA IgA reactive and villous atrophy in small-bowel biopsies) was 3.4% among 172 individuals with autoimmune thyroid disease.^42^ Mainardi et al. demonstrated that the prevalence of celiac disease (IgG/IgA AGA, IgA TTG and IgA EMA antibodies and villous atrophy in duodenal biopsies) was 2% among 100 patients with autoimmune thyroiditis. The serological markers became undetectable six months after a gluten-free diet was started. However, thyroid autoantibodies did not positively correlate with dietary habits.^43^ Volta et al. showed that there was higher prevalence of celiac disease (IgA TTG and/or IgA EMA, villous atrophy and intraepithelial lymphocytes > 40%) among 220 individuals with autoimmune thyroiditis than in 250 blood donors (3.2% versus 0.4%; P = 0.022).^44^

Both celiac disease and autoimmune thyroiditis are more common among women.^21^^,^ 34 ^,^ 41 ^,^ 42 ^,^ 44 This is consistent with the findings in the present study, in which 92.5% of the patients with autoimmune thyroiditis were female. However, the proportion of women found among the patients who were serologically positive for celiac disease was 3:2 (60%), which is similar to what was previously described by Ravaglia et al. (63%),^19^ and lower than what was reported by Collins et al. (71%).^34^

Even though the patients with autoimmune thyroiditis included in this study presented ages between 19 and 74 years old, four individuals with celiac disease were aged under 25. A wide age range at the time of diagnosing celiac disease among patients with autoimmune thyroiditis has been described, with mean ages ranging from 54.7 to 60.3 years (overall age range: 26-84).^19^^,^ 42 ^,^ 44 Although it has been reported that the risk of celiac disease in patients with autoimmune thyroiditis increases with age,^19^ it is known that celiac antibodies appear earlier than histological and clinical features.^24^ Celiac disease was traditionally considered to be a childhood disease, but most patients are now diagnosed in adulthood. It remains unclear why the prevalence of celiac disease seems to have increased over time.^10^ A prevalence of celiac disease of 0.1% was demonstrated among elderly Brazilians.^45^ Despite the classic presentation in this group, the diagnosis is often delayed because of investigations for colonic neoplasia to explain diarrhea, anemia, weight loss and weakness.^46^

In the present study, the median BMI did not differ between subjects with positive and negative serological tests, and both groups had a median BMI greater than 25 kg/m^2^, which classified them as overweight. Although weight loss is a recognized presentation of celiac disease,^6^^,^ 7 a substantial proportion of patients are overweight.^47^ Among 371 individuals with biopsy-proven celiac disease, Dickey et al.^47^ showed that 39% were overweight according to the World Health Organization criteria,^48^ and 13% were obese.^47^ This finding could reflect the absence of clinical manifestations in serologically positive patients (celiac disease in a latent form).^10^^,^ 16 Overweight is frequently described as a clinical feature of hypothyroidism; nevertheless, the TSH levels were similar between the two groups (P = 0.351) and the median TSH remained within the normal range, thus suggesting that the disease was compensated in most of the subjects included. This characteristic probably reflects the worldwide trend towards increasing weight, secondary to lifestyle.^49^ Between 1975 and 1979, the prevalence of obesity in Brazil increased by 92% for men and 63% for women. Between 1989 and 2003, obesity rates remained stable among women.^50^ The prevalence of obesity reported among Brazilians ranges from 5% to 19%,^51^^-^ 53 while in the United States, the prevalence of obesity is 32% among adults, which is much higher than reported elsewhere.^54^ In that country, Cheng et al. evaluated 369 individuals with celiac disease and showed that 15.2% of them presented overweight and 6.8% were obese.^55^

Diarrhea is the most common clinical manifestation of celiac disease; however, it is present in less than 50% of the cases.^8^^-^ 10 In the present study, 20% of the individuals who were serologically positive for celiac disease presented diarrhea, and its prevalence did not differ between the two groups. Since this was a screening study, it was expected that individuals who were serologically positive for celiac disease would mostly be asymptomatic. Among the studies that evaluated the prevalence of celiac disease in patients with autoimmune thyroid disease, most patients did not show symptoms.^37^^,^ 41 ^,^ 42

Serum ferritin is a secretory component synthesized from intracellular ferritin, which reflects an individual's iron reserves. Hypoferritinemia can be found in cases of iron deficiency, through decreased inventories of this mineral, and also in cases of scurvy and hypothyroidism. In the latter, it occurs through decreased synthesis of serum ferritin. High iron stores are associated with higher levels of plasma ferritin, but hyperferritinemia may also be observed in states of inflammation, cancer, tissue damage and increased metabolism, which make proteins sensitive to but not very specific for assessing the iron balance.^56^ Transferrin is the principal iron transport protein in the extracellular compartment. When there is a large amount of stored iron, there is less transferrin production, and its saturation reflects the iron stores.^57^ Among 13 individuals with autoimmune thyroiditis and celiac disease, Meloni et al. observed hypoferritinemia in 46% of them.^32^ In the present study, all the individuals who were serologically positive for celiac disease presented hypoferritinemia. In comparison with the other individuals, the serum levels of ferritin were lower, but this difference was not statistically significant. Rather, the hemoglobin level was higher among serologically positive individuals, possibly because the proportion of men was higher, given that men are known to have higher hemoglobin levels than women.^57^

Future studies should compare celiac disease prevalence between healthy individuals and those with autoimmune thyroid disease. It would also be of great interest to evaluate the influence of a gluten-free diet on thyroid disease treatment and TSH control, among individuals with both diseases.

## CONCLUSION

The prevalence of celiac disease among patients with autoimmune thyroid disease was 9.3%. One person complained of diarrhea and none of the patients presented anemia. In comparison with other individuals, the group of patients who were serologically positive for celiac disease presented lower median age, a lower proportion of females and higher median hemoglobin levels. In at-risk populations, such as patients with autoimmune thyroid disease, signs and symptoms such as anemia and diarrhea should not be used as criteria for celiac disease screening. Celiac antibodies ought to be tested in all subjects with autoimmune thyroiditis, because of its high prevalence.
